# ATP-binding cassette transporter 13 mRNA expression level in schizophrenia patients

**DOI:** 10.1038/s41598-020-78530-9

**Published:** 2020-12-09

**Authors:** Lu Qian, Yu Qin, Xinyu Chen, Fuquan Zhang, Bixiu Yang, Kunlun Dong, Zhiqiang Wang, Kai Zhang

**Affiliations:** 1 Department of Psychiatry, Wuxi Mental Health Center, Nanjing Medical University, 156 Qianrong Road, Wuxi, 214151 China; 2 Department of Psychiatry, Chaohu Hospital of Anhui Medical University, 64 North Chaohu Road, Hefei, 238000 China; 3 Anhui Psychiatric Center, Anhui Medical University, Hefei, China

**Keywords:** Schizophrenia, Psychiatric disorders

## Abstract

The objective of this study was to investigate the expression and clinical role of ATP-binding cassette transporter 13 (ABCA13) gene previously shown to be associated with schizophrenia (SZ) through Genome-wide association studies studies. Thirty-two first-episode drug-naive SZ patients and forty-eight age and gender-matched healthy controls were enrolled in this study. We measured ABCA13 mRNA expression levels using quantitative real-time PCR at baseline and 12 weeks after antipsychotic therapy. Moreover, clinical symptoms were measured by the Positive and Negative Syndrome Scale (PANSS) at baseline and 12-week follow-up. We found that ABCA13 mRNA levels were significantly lower in SZ patients compared with healthy controls at baseline. SZ patients’ symptoms were decreased, but ABCA13 mRNA levels were increased after 12 weeks antipsychotic therapy. In addition, there was a significant difference in ABCA13 mRNA levels among SZ patients at baseline and 12-week follow-up. The ABCA13 mRNA levels were not associated with age, BMI, years of education. Of the clinical symptoms measured, the ABCA13 mRNA levels were negatively associated with the PANSS scores at baseline and 12-week follow-up. The results indicated that the ABCA13 mRNA expression level is of interest, and upon further studies, it could be used as a biomarker for SZ treatment outcome.

## Introduction

Schizophrenia (SZ) is a severe psychiatric disorder and characterized by a combination of psychotic symptoms. According to Huang’s epidemiological survey, the lifetime prevalence of SZ in China is about 0.7%^1^. About 9.8 million patients are suffering from SZ in China. It is well-known that SZ imposes a massive burden on individuals, their families, and healthcare providers^2^. However, the etiology of SZ is not clear yet. Previous studies have shown that the heritability of SZ is 60–85%^3,4^. Among various hypothesis for SZ’s pathogenesis, genetics factors are gradually supposed to play more significantly roles. As a result, numerous genome-wide association studies (GWAS) and other studies have identified and validated several possible candidate genes contributing to SZ. Among these SZ candidate genes, ATP-binding cassette transporter 13 (ABCA13) recently received increasing attention^5–8^.

ABCA13, located on human chromosome 7p12.3, is a member of the ATP-binding cassette super-family encoding transporters shuttling various substrate across cellular membranes^9^. ABCA13 is widely expressed in the human brain, bone marrow, and other tissues^10^. A study indicated that the expression of ABC transporters was vital to maintain the equilibrium of lipids, peptide, and small molecules within the compartment, and was vital for normal brain function^11^. Previous studies in both macaque and drosophila models have shown that the ABCA13 gene is strongly associate with neurodevelopment, and that deletion of the gene leads to autism-like behavior in these animals^12–14^.

Knight and colleagues revealed that disruption of ABCA13 expression in human hippocampus and frontal cortex implicated aberrant lipid biology as a pathological pathway in mental illness^5^. However, the functional role of ABCA13 at the level of gene expression and relationship to the severity of core psychotic symptom of SZ is still unknown.

Based on the results of previous studies, we proposed our hypothesis. Patients with SZ have abnormal ABCA13 expression. Treatment with antipsychotic drug results in the relief of psychotic symptoms and restoration of the abnormal ABCA13 gene expression.

To our knowledge, this is the first study to explore the mRNA expression level of ABCA13 in peripheral blood by RT-qPCR in the Han Chinese population. Therefore, this study is an essential supplement to previous studies and maybe a new area for future research of SZ.

## Materials and methods

### Participants

Forty drug-naive SZ patients were enrolled from Wuxi Mental Health Center, Nanjing Medical University. The inclusion criteria for the patient group in our study were: (1) current SZ according to the Diagnostic and Statistical Manual of Mental Disorders, 5th edition (DSM-5); (2) Han Chinese, between 18 and 65 years old; (3) had not received anti-psychiatric drugs more than 2 weeks. SZ patients were excluded if they met any of the following exclusion criteria: (1) met the DSM-5 criteria for other mental illnesses, such as major depressive disorder, obsessive–compulsive disorder, and so on; (2) present use of antipsychotic drugs; (3) a serious organic disease in the last 3 months; (4) pregnant and lactating women. The enrolled patients received risperidone monotherapy, and reached a therapeutic dose (4–8 mg/d) within 2 weeks.

Forty-eight healthy controls were recruited from the staff and doctors of Wuxi Mental Health Center, Nanjing Medical University. All healthy controls were Han Chinese, and had no self-reported family history of psychiatric disorders or drug use history.

This study was approved by the Ethics Committee of Wuxi Mental Health Center, Nanjing Medical University (NO. WUXIMHCIRB2019-006). In addition, all the study procedures were in line with the Declaration of Helsinki. All participants wrote informed consent after a detailed description of the study.

### Socio-demographic, clinical characteristics, and psychiatric symptoms

Socio-demographic characteristics were collected from all participants, such as gender, age, marital status, and education. Positive and Negative Syndrome Scale (PANSS) was used to measure the psychotic symptoms of SZ patients. The PANSS scale was assessed by a senior psychiatrist at baseline and 12 weeks after drug therapy, and the intra-class correlation coefficient between the four raters was greater than 0.8. The procedure of PANSS assessment was performed as previously reported^15,16^.

### RNA isolation and expression analysis

Five milliliter fasting elbow venous blood samples were collected from the healthy controls and the SZ patients at baseline. We also collected another 5 ml blood samples from the SZ patients after 12 weeks drug therapy. Total RNA was isolated from peripheral blood mononuclear cells (PBMCs) using Trizol reagent (Invitrogen, CA, USA) as previously reported^17^. cDNA was synthesized using High Capacity RNA-to-cDNA Kit (Invitrogen; USA) as described by the manufacturer. RT-qPCR was performed using the primers Forward: TGCGAGTCTACCAACAGGTG and Reverse: TTATCTTTTGGGCCATCTGC, and a SYBR Select Master Mix (Invitrogen, CA, USA). PCR was performed using a 7900HT real-time PCR machine (Applied Biosystems, USA) for 2 min at 50 °C, 2 min at 95 °C, and then 40 cycles consisting of 15 s at 95 °C, 60 s at 60 °C, followed by a subsequent standard dissociation protocol to ensure that each amplicon was a single product. All quantifications were normalized to ACTB. The RT-qPCRs were performed in triplicate for each of the three independent samples. Fold change in mRNA levels between cases and controls was calculated using 2^−ΔΔCt^ method.

### Statistical analysis

The data were expressed as the mean ± standard deviation. We used the Student’s t test to examine the differences of various numerical variables between the SZ group and the healthy control group. The Mann–Whitney U test was used to compare non-normal distributions of variables. Classified variables were compared by Chi-square test. We compared the ABCA13 levels between the patients and the healthy controls at baseline and at 12 weeks using one-way analysis of variance (ANOVA), followed by post hoc Fisher’s Least Significant Difference (LSD) test. Pearson correlation analysis was used for correlation analysis. All statistical analyses were performed using statistical software (PASW Statistics 18, Chicago, IL, USA). A *P* value of < 0.05 was considered statistically.

## Results

### Socio-demographic characteristics and evaluation of clinical symptoms

The socio-demographic data of SZ patients and healthy controls are presented in Table 1. In this study, we enrolled 40 patients with SZ and 48 healthy controls. However, only 33 SZ patients finished the PANSS assessment after 12 weeks drug therapy. There were no significant differences in age, gender, and education between the two groups (all *P* values > 0.05) (Table 1). In the SZ patient group, the PANSS total score, positive scale score, negative scale score, and general psychopathology scale score at 12 weeks after drug therapy were significantly lower than these scores at baseline (all *P* values < 0.05) (Table 1).

**Table 1:** Socio-demographic data of patients with schizophrenia and healthy controls.

	Healthy controls (n = 48)	SZ patients		t/χ^2^	*P*
		Baseline (n = 40)	Week 12 (n = 33)		
Age (years)	38.98 ± 10.07	38.55 ± 10.10.90		0.192	0.848
Gender (male)	29 (60.41)	24 (60.00)		0.73	0.39
Education (years)	10.90 ± 2.51	11.00 ± 2.72		− 0.19	0.85
**PANSS**					
Total score		101.40 ± 13.87	65.85 ± 16.04	10.16	0.00
Positive scale		21.93 ± 4.63	11.33 ± 3.89	10.45	0.00
Negative scale		25.20 ± 7.92	19.18 ± 5.78	3.64	0.02
General psychopathology scale		47.08 ± 7.18	32.12 ± 7.38	8.75	0.00

*SZ* schizophrenia, *PANSS* Positive and Negative Syndrome Scale.

### ABCA13 mRNA expression levels in the healthy controls and the SZ patients at baseline and 12-week follow-up

**Figure 1:**
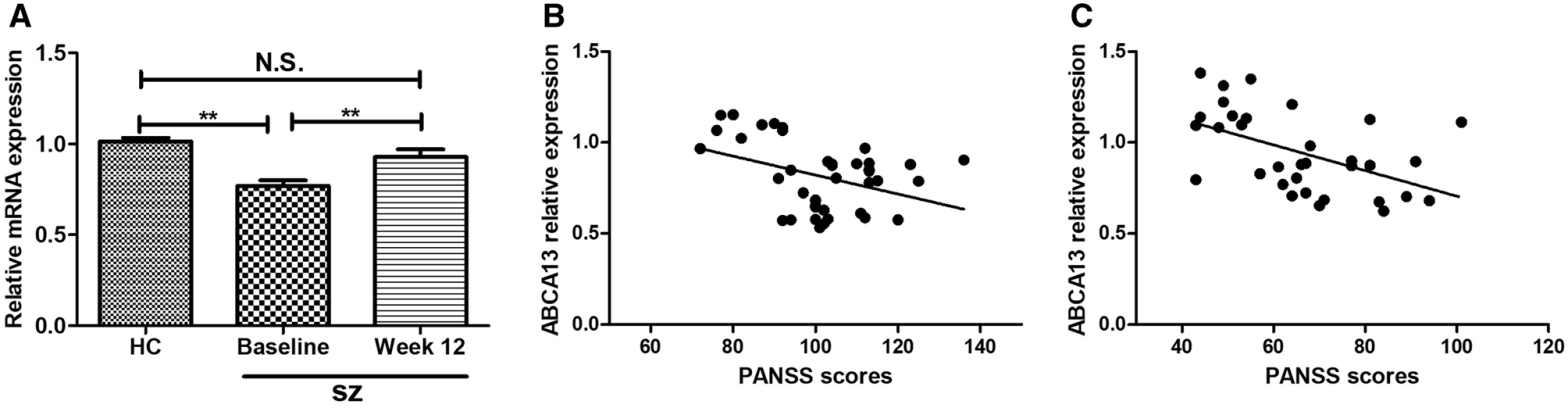
(**A**) ABCA13 relative expression levels in the healthy controls and the SZ patients at baseline and 12-week follow-up. (**B**) Scatter plot and regression line of ABCA13 relative expression levels and PANSS scores at baseline. (**C**) Scatter plot and regression line of ABCA13 relative expression levels and PANSS scores at 12-week follow-up. *HC* healthy controls, *SZ* schizophrenia, *PANSS* Positive and Negative Syndrome Scale. **P < 0.01.

The ABCA13 mRNA expression levels are shown in Fig. 1A. The result indicated a difference among all participants (F = 12.92, *P* < 0.01). The mean ABCA13 mRNA expression levels of SZ patients were significantly lower at both baseline (0.81 ± 0.19) and after 12 weeks of drug therapy (0.95 ± 0.22) compared to the healthy controls (1.01 ± 0.15). The ABCA13 mRNA expression level increased after 12 weeks of anti-psychiatric drug therapy than baseline (*P* < 0.05).

### Correlations between ABCA13 mRNA levels, demographic factors, and clinical symptoms

The ABCA13 mRNA levels were not associated with age, BMI, and education (data not shown). Of the clinical symptoms measured, there was a negative association between ABCA13 mRNA relative expression levels and PANSS total scores at baseline (r =  − 0.38, *P* < 0.05, see Figurer 1B for details). After 12 weeks drug therapy, there was a negative association between ABCA13 mRNA relative expression and PANSS scores too (r =  − 0.51, *P* < 0.01, see Fig. 1C for details). However, the change in ABCA expression levels from baseline to 12-week follow-up was no negatively associated with the change in PANSS scores (data not shown).

## Discussion

In this study, we found lower ABCA13 mRNA levels in the SZ patients compared to healthy controls. The result of our study supports the notion that ABCA13 might play a critical role in SZ. ABCA13, the largest member of the ABCA protein family, is widely expressed in the human hippocampus, cortex, blood, and other tissues. Based on the structure of ABCA13, it has been suggested that it is involved in the transport lipid molecules across cell membranes. However, its precise function remains unclear. Some scientists have focused on the relationship between ABCA13 and psychiatric disorders. Yoshida and colleagues reported a mutation Japanese macaque with spontaneously autism-like symptoms, like impaired social ability, restricted and repetitive behaviors^18^. After whole-exome sequencing and copy number variation analyses, they identified rare coding variants of ABCA13 in the Japanese macaque. Since then, researchers have found that the ABCA gene knocked out flies show autism-like symptoms^14^. Monkey with ABCA13 deletion also showed similar symptoms of autism^12^.

A study from Knight and colleges reported that rare genetic variants in ABCA13 increase susceptibility to schizophrenia^5^. In contrast, other studies have indicated that there was no association between copy number variants in ABCA13 and schizophrenia^19–21^.

In addition to rare genetic variants, some researchers have also explored the relationship between common genetic variants in ABCA13 and the pathogenesis of schizophrenia. Tomioka et al. reported that a single nucleotide polymorphism (SNP) (T4031A) in ABCA13 affect the function of ABCA13 in the brain, and might concern neurological disorder^22^. Chen and colleagues analyzed tag SNPs of ABCA13 in Han Chinese patients with schizophrenia and healthy controls^23^. They found that rs17132388 and rs6583476 of ABCA13 show a statistically significant association with schizophrenia. ABCA13 gene may contain genetic risk factors for schizophrenia in the Han Chinese population. In addition, Ma et al. also designed a case–control study to examine whether the common variants of ABCA13 gene were associated with schizophrenia in a Han Chinese sample^24^. Different from Chen’s study, they enrolled 488 unrelated patients with schizophrenia and 506 healthy controls from the Northwestern region of China. In Ma’s study, the allele frequencies and the genotype distributions of rs6955686 showed a nominal association between schizophrenia patients and controls. However, the significant differences did not survive Benjamini correction. Considering the interactions between rare and common variants, further genetic and functional studies of ABCA13 are necessary to elucidate its possible role in schizophrenia.

Rajkumar et al. identified 12 novel differentially expressed genes including ABCA13 in patients’ postmortem brains with Lewy body dementias (LBD) through genome-wide statistical significance^25^. ABCA13 expression levels were down-regulated after patients suffer from LBD. To our knowledge, our study is the first study to explore the mRNA expression level of ABCA13 in schizophrenia. We found that ABCA13 mRNA levels were significantly lower in schizophrenia patients compared with healthy controls at baseline. ABCA13 is the largest member of the ABC protein family. It is proven to be expressed mainly in the human brain and other tissues. ABCA13 acts as a shuttle mediator across cell membranes of lipids to transport lipid molecules, and play a role in maintaining brain lipid homeostasis^26^. Down-regulation of ABCA13 expression affects lipid metabolism and leads to abnormal lipid metabolism in patients with schizophrenia. In fact, the malfunctions of lipid metabolism have been found in patients with schizophrenia in our previous studies^16,27^. In this study, we also found that ABCA13 mRNA levels were increased after 12 weeks antipsychotic therapy. This result suggests that abnormal lipid metabolism in treated patients is expected to return to normal.

The ABCA13 mRNA levels in our study were negatively associated with the PANSS scores at baseline and 12-week follow-up. No related literature was found to help us to explain this finding. More studies are needed to look at the association between ABCA13 and psychiatric symptoms.

There were several limitations in our study. First, the sample size of our study was small. Only 40 SZ patients were included in this study. In order to be able to make the results more convincing, we will enroll more samples in future studies. Second, our study only measured the expression of ABCA13 mRNA from the patients' peripheral blood. More tissues from more sites need to be collected in future studies to validate our results, such as brain tissue. Third, this study was not done to test for gene polymorphisms. Gene polymorphisms may affect gene expression.

In summary, our results show that the ABCA13 expression levels in SZ patients are lower than those of healthy controls at baseline, and increased after 12 weeks of drug therapy. However, the ABCA13 expression levels are still lower than those of the healthy controls. Interestingly, there was a negative correlation between ABCA13 expression levels and psychiatry symptoms in our study. The results indicated that the ABCA13 mRNA expression level is of interest, and upon further studies it could be used as a biomarker for SZ treatment outcomes.
